# Left ventricular longitudinal strain in soccer referees

**DOI:** 10.18632/oncotarget.15242

**Published:** 2017-02-09

**Authors:** Luigi Gianturco, Bruno Bodini, Vincenzo Gianturco, Giuseppina Lippo, Agnese Solbiati, Maurizio Turiel

**Affiliations:** ^1^ IRCCS Galeazzi Orthopedic Institute, Cardiology Unit, Milan, Italy; ^2^ IRCCS Galeazzi Orthopedic Institute, Rehabilitation Unit, Milan, Italy; ^3^ Foro Italico University of Rome, Rome, Italy; ^4^ Department of Occupational and Environmental Health University of Milan, IRCCS Fondazione Policlinico Mangiagalli Regina Elena, Milan, Italy

**Keywords:** soccer refereeing, VO2max, speckle tracking echocardiography, myocardial function, LV mechanics

## Abstract

Along the years, the analysis of soccer referees perfomance has interested the experts and we can find several types of studies in literature using in particular cardiac imaging. The aim of this retrospective study was to observe relationship between VO2max uptake and some conventional and not-conventional echocardiographic parameters. In order to perform this evaluation, we have enrolled 20 referees, belonging to Italian Soccer Referees' Association and we have investigated cardiovascular profile of them. We found a strong direct relationship between VO2max and global longitudinal strain of left ventricle assessed by means of speckle tracking echocardiographic analysis (R2=0.8464). The most common classic echocardiographic indexes have showed mild relations (respectively, VO2max vs EF: R2=0.4444; VO2max vs LV indexed mass: R2=0.2268). Therefore, our study suggests that longitudinal strain could be proposed as a specific echocardiographic parameter to evaluate the soccer referees performance.

## INTRODUCTION

Leading soccer federations include into their organisation specialists for monitoring of referees' health and performance. In literature, in the last two decades, we can find an increasing number of articles about soccer refereeing [1, 2]. Castagna *et al*. [3] extensively reviewed physiological aspects of referees; moreover, specific scientific background applied to soccer referees has been developed in last years [4].

In the last few years, some papers focused the attention on perceptual-cognitive expertise [5]. However, the experts also studied physiological characteristics of referees. Anthropometric measurements have been usually collected and analyzed together with maximal oxygen uptake (VO2max). Aerobic and anaerobic responses were acquired by means of specific fitness tests. Among them, we can quote Yo-Yo Intermittent recovery test level 1 (YY-IR1) which is frequently used by some national referees associations including Italian Referees' Association [6]; YY-IR1 has substituted the previous Cooper test cited in the first studies on Italian referees [7].

Refereeing is substantially an endurance sport and therefore, along the last years there was a continuous evaluation of novel and/or efficient parameters to assay referees' performance. Knowledge of heart physiology and cardiovascular (CV) system is fundamental to investigate fitness performance and to select the most indicated tools as well as echocardiography that is increasing in importance [8, 9]. Many data may be achieved performing echocardiographic exams and linked to other indexes of CV function such as VO2max [10]. Furthermore, echocardiograhic studies are not invasive and quite cheap; then, they are very useful tools in order to investigate physical performance of athletes, even atypical, as referees.

Generally, prolonged and intensive exercise modifies heart by increasing of mass, wall thickness and diameters of chambers. These changes of LV may differ in relation to the type of sport, body size, sex and race. Refereeing is properly long-time, continuous and intensive activity during a period of about 20-30 years; echocardiography is also pivotal for preventing hypertrophic heart diseases [11]. Moreover, as demonstrated by Castagna *et al*. soccer referees run on average about 12 km during each match [1–3] and it is known that the high and continuously workload of soccer referees is a potential risk factor for developing of pathological heart hypertrophy [12].

Nowhere, in addition to classic panel of echocardiographic measurements we may use some novel techniques such as speckle tracking echocardiograhy (STE) which gives us a further instrument in left ventricle (LV) study [13, 14]. Moreover, in athletes, conventional echocardiography very often shows normal systolic and diastolic functions assessed by means of ejection fraction (EF) evaluation and Doppler/Tissue Doppler Imaging (TDI) [15]. So, in the past few years new techniques have been introduced to clarify the intrinsic myocardial performance and not only for assessing LV systole and diastole. Among them, deformation imaging by two-dimensional (2D) STE has been shown to provide more specific information beyond EF and in early stages of pathologic scenario [16].

Finally, the aims of our present study were to define the characteristics of LV myocardial mechanics as assessed by 2D STE in a group of top level referees and to correlate those findings with a traditional marker of aerobic power such as VO2max.

## METHODS AND MATERIALS

We have consecutively studied 20 soccer referees, belonging to Italian Referees' Association. All of them were males aged 27 to 43 (mean 33.87±9.56). No one had hystory of heart disease.

The subjects underwent a physical examination, a routinary 12-leads electrocardiography (ECG), an echocardiographic exam and a VO2max assessment by means of cardio-pulmonary exercise testing (CPET).

All athletes agreed to take part in the study and provided written informed consent; the study protocol was approved by the institutional review board.

### Echocardiography

Echocardiographic examinations were performed by expert cardiologists with specific competence in the evaluation of athletes, using a commercial ultrasound machine (iE33; Philips Medical System, Andover, USA) equipped with an S5 probe (1-4 Mhz). 2D measurement of LV cavity diameters, wall thickness, mass were assessed according to American Society Echocardiography criteria [17].

LV EF were measured with the biplane Simpson's rule from the apical four- and two-chambers views, while LV mass was measured with Devereux's formula [18]. The Doppler indices of LV diastolic function were measured using standard techniques [19].

### Speckle tracking analysis

Analysis of 2D strain imaging was performed offline with commercially available software (QLAB version 10.0; Philips Medical Systems, USA). This latest version was developed following the recommendation of a joint American Society of Echocardiography and American Heart Association document, aimed to improve standardization of the strain analysis and reduce intervendor variability [20].

Apical four-chamber views were acquired achieving an high frame rate (70-80 frames/sec) and fitting the entire LV in the echocardiographic plane [21].

Attention was paid to acquire cardiac cycles of the same standardized length and during the same respiratory phase (expiration); so, three cardiac cycles were stored in cine-loop format for off-line analysis in order to assess end-systolic LV longitudinal strain. The endocardial border was traced on an end-diastolic frame and subsequently automatically tracked; the tracking was verified in real time and corrected by adjusting the region of interest or manually correcting the border. End-systole was identified as corresponding to aortic valve closure as measured by pulsed-Doppler.

The software represents myocardial deformation in the form of time-strain graphs in which it is possible to identify the different phases of the cardiac cycle as follows: a negative wave is observed during systole, which reaches its negative peak at the time of aortic valve closure and represents maximal longitudinal myocardial shortening during contraction; during diastole, the strain values progressively increase towards the original length.

The time-strain curves were obtained and analysed by two independent observers who were blinded to the clinical data. Inter-observer variability was calculated using the Bland-Altman method to compare the measurements made by the two observers in 10 randomly selected subjects [22], and was <5% for global longitudinal strain (GLS).

### Cardiopulmonary exercise testing

All the referees enrolled were submitted to a treadmill CPET. Expired gases were analyzed using a commercial gas analyzer (Quark b2, Cosmed, Pomezia, Italy) that enabled VO2max data collection. Gas and HR sampling were performed in continuous as established by EACPR/AHA Position Paper [23].

### Statistical analysis

Data were expressed as mean ± standard deviation (SD). Relations with variables of interest were detected using simple linear regression (r).

In a second phase, a Kruskal-Wallis ANOVA one way test was performed to assess how and which variables may better affect VO2max.

Significance was set for the calculations at 5% (*p*≤0.05).

The free statistics software by Patrick Wessa was used for computations [24].

As mentioned, inter-observer variability was calculated using the Bland-Altman method to compare the measurements made by the two observers in 10 randomly selected subjects.

## RESULTS

The main characteristics of the study population are reported in Table 1: there are physiological values of body mass index (BMI) and blood pressure values; moreover, heart rate (HR) was in reference ranges, while VO2max is comparable with values acquired in already quoted studies about referees [1–4]. No morphological abnormalities were found in ECGs.

**Table 1:** Basal characteristics of population

Parameters	Means (±SD)
Age (years)	33.87 (9.56)
Height (m)	1.86 (0.05)
Weight (Kg)	77.20 (4.37)
BMI (Kg*m^−2^)	22.40 (0.80)
SBP (mmHg)	124.33 (9.80)
DBP (mmHg)	76.00 (6.04)
HR (beats*min^−1^)	61.93 (3.84)
VO_2max_ (ml*kg^−1^*min^−1^)	50.39 (0.70)
Number of training sessions/year	196.55 (7.10)

Data are presented as means (±SD). BMI, body mass index; SBP, systolic blood pressure; DBP, diastolic blood pressure; HR, heart rate; VO_2max_ = maximal oxygen uptake.

Table 2, instead, shows routinary echocardiographic parameters assessed. Referees showed normal values of diameters, volumes and cardiac mass within reference range. Respectively, left ventricular end-diastolic diameter mean (LVEDD) was 52.95±2.04mm, left ventricular end-systolic diameter (LVESD) 33.35±2.25mm, left ventricular end-diastolic volume (LVEDV) 115.80±2.69ml, left ventricular end-systolic volume (LVESV) 40.55±3.94ml and finally the indexed mass of LV 109.75±5.99g/m^2^.

**Table 2:** Echocardiographic parameters

Parameters	Means (±SD)
LVEDD (mm)	52.95 (2.04)
LVESD (mm)	33.35 (2.25)
LVEDV (ml)	115.80 (2.69)
LVESV (ml)	40.55 (3.94)
EF (%)	64.97 (4.88)
LV indexed mass (g*m^−2^)	109.75 (5.99)
GLS (%)	23.34 (3.73)

Data are presented as means (±SD). LVEDD, left ventricular end-diastolic diameter; LVESD, left ventricular end-systolic diameter; LVEDV, left ventricular end-diastolic volume; LVESV, left ventricular end-systolic volume; EF, ejection fraction; LV indexed mass, left ventricle indexed mass; GLS, global longitudinal strain, expressed as usual as negative value.

Findings derived from statistical analysis are shown in Table 3 where VO2max is identified as dependent variable and compared with various indexes such as diameters, volumes, LV mass, EF, GLS and workloads in term of total number of training sessions during a year. We may appreciate a statistical significativity for EF, volumes, LV mass but especially for GLS.

**Table 3:** ANOVA Kruskal-Wallis one way analysis

ANOVA Kruskal-Wallis one way test VO_2max_ (dependent variable)	
Parameters	*P*
LVEDD (mm)	NS
LVESD (mm)	NS
LVEDV (ml)	.05
LVESV (ml)	.05
EF (%)	.03
GLS (%)	.002
Number of training sessions/year	NS
LV indexed mass (g*m^−2^)	.04

P values are shown. NS=not significative. LVEDD, left ventricular end-diastolic diameter; LVESD, left ventricular end-systolic diameter; LVEDV, left ventricular end-diastolic volume; LVESV, left ventricular end-systolic volume; EF, ejection fraction; GLS, global longitudinal strain, expressed as usual as negative value; LV indexed mass, left ventricle indexed mass.

Intra- and inter-observer variability did not affect the data interpretation.

**Figure 1:**
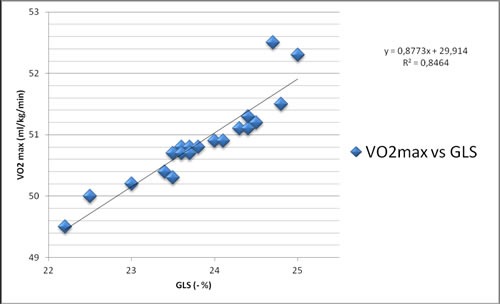
Correlation between VO2max and GLS

**Figure 2:**
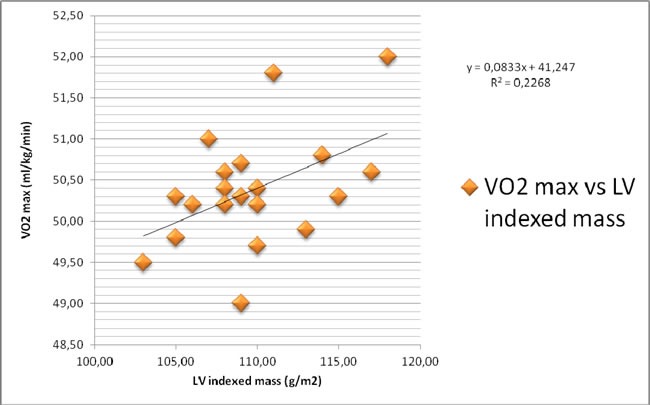
Correlation between VO2max and LV indexed mass

In Figure 1 the correlation between VO2max and GLS is pictured. In Figure 2 the correlation between VO2max and LV indexed mass is represented.

A significant correlation exists between VO2max and LV indexed mass, while a very significant correlation is between VO2max and GLS.

## DISCUSSION

Heart morphological modifications induced by physical exercise have been extensively studied in the past decades: a specific physiopathological condition known as “athletes’ heart” has been identified [25].

In the last years, the focus has moved on functional remodelling of LV of athletes [26]. So, STE has been becoming pivotal for assessing training-induced changes in athlete's heart [27]. The growing application of STE as novel imaging technique is motivated by its higher efficiency in analyzing twist mechanics of LV and/or in diagnosis of cardiomyopathies and/or different cardiac prolonged exercise-induced abnormalities [28]. Then, STE is able to detect subclinical modifications even before overt LV hypertrophy [29].

In literature, we have found some papers inhering the study of soccer refereeing [1–4]. The focus of different studies was variable. However, some authors tried to define an echocardiographic profile of referees [30, 31] in order to clarify physical and physiological items related to refereeing activity and to prevent pathological modifications of cardiac structure induced by long-time and high workload exercise.

About the right use of imaging in cardiology, nowhere the multi-modalility approach is more and more important to reach better findings in clinical pratice as recently stated [32]. Otherwise, in sports medicine, it should be desirable a more habitual integrated use of different tools to investigate athletes' performance and post-processing imaging software such as speckle tracking techniques [33]. The informations achieved with several type of instruments may be used all together in order to improve global clinical assessment and to obtain better outcomes and follow-up. Among all possible imaging exams, we have chosen the most comfortable assessment such as echocardiography (2D and STE) because of low costs and not-invasive characteristics.

Therefore, in our study traditional echocardiography provided traditional panel of parameters as already reported in previous studies. Then, we focused the attention on speckle tracking assessments and they also were in reference ranges, such as in other studies conducted on professional soccer players [34].

So we investigated main usual 2D parameters and then we have focused our attention on STE. LV mass (p=0.04) and EF (p=0.03) showed a weak relation with VO2max. Total number of training sessions/year were not similarly related to VO2max.

Moreover, as already demonstrated by Urbano-Moral *et al*. it is tipical to discover athlete's heart with a normal EF in which hypertrophy is growing and modifying longitudinal deformation capability [35].

Then, the best marker related to adjustments exercise-induced seem to be GLS and in fact, the best relation observed for VOmax was with GLS assessed with STE (p=0.002).

Therefore, this innovative technique provides clinical and functional data which can efficiently differentiate physiological status and pathological one. For several years, we used only conventional echocardiography to measure exercise effects on cardiac morphology/function and diameters, volumes, EF and LV mass but the technology was not able to detect early functional adaptations of athletes' heart. Today, instead, we recommend to systematically apply STE and its derived indexes that are recognized to be important in the characterisation of myocardial properties and/or in distinguishing “athletes' heart” and cardiomyopathies.

We know that all tracking techniques are more robust and reproducible for global rather than regional assessments. Moreover, GLS averaged from the apical views is the most important and reproducible of all LV deformation parameters and has been shown to be a powerful diagnostic and prognostic tool. This last consideration was also recently confirmed by recommendations for cardiac chambers quantification [36].

However, literature has not totally clarified which determinants might potentially influence LV strain and so, GLS. In fact, it is still controversial if and how demographic features (including gender), haemodynamic factors and cardiac dimensions could affect GLS [37, 38].

Different type of sports might also influence strain parameters as investigated by Caselli *et al*. [39].

In any case, although the clear identification of items affecting LV strain measurements is still debated our findings confirm the emerging and promising role of STE for LV mechanics investigation in endurance athletes.

## CONCLUSIONS

In conclusion, this study confirmed the importance of imaging, especially echocardiography, for the assessment of athletes' heart performance in endurance athletes, as soccer referees, and seems to suggest STE as the echocardiographic tool most able to detect early modifications in LV mechanics before of conventional transthoracic echocardiography parameters. The main explanation could be identified in specific modality of STE which is a technique very very close to Torrent-Guasp model describing LV mechanics [40, 41].

Finally, STE should become one of the most important approaches for studying sports' men, including soccer referees, and monitoring physical performance and possible induced pathophysiological mechanisms underlying sport activity along and for several years.
